# The spatial ecology of free-ranging domestic pigs (*Sus scrofa*) in western Kenya

**DOI:** 10.1186/1746-6148-9-46

**Published:** 2013-03-07

**Authors:** Lian F Thomas, William A de Glanville, Elizabeth A Cook, Eric M Fèvre

**Affiliations:** 1 Centre for Immunity, Infection and Evolution, Institute for Immunology and Infection Research, School of Biological Sciences, University of Edinburgh, Ashworth Laboratories, West Mains Road, Edinburgh EH9 3JT, UK; 2 International Livestock Research Institute, Old Naivasha Road, PO Box 30709, 00100 Nairobi, Kenya

**Keywords:** Pig, GPS, *Taenia*, *Trichinella*, African swine fever, *Toxoplasma*, *Trypanosoma*, Free range

## Abstract

**Background:**

In many parts of the developing world, pigs are kept under low-input systems where they roam freely to scavenge food. These systems allow poor farmers the opportunity to enter into livestock keeping without large capital investments. This, combined with a growing demand for pork, especially in urban areas, has led to an increase in the number of small-holder farmers keeping free range pigs as a commercial enterprise. Despite the benefits which pig production can bring to a household, keeping pigs under a free range system increases the risk of the pig acquiring diseases, either production-limiting or zoonotic in nature. This study used Global Positioning System (GPS) technology to track free range domestic pigs in rural western Kenya, in order to understand their movement patterns and interactions with elements of the peri-domestic environment.

**Results:**

We found that these pigs travel an average of 4,340 m in a 12 hr period and had a mean home range of 10,343 m^2^ (range 2,937–32,759 m^2^) within which the core utilisation distribution was found to be 964 m^2^ (range 246–3,289 m^2^) with pigs spending on average 47% of their time outside their homestead of origin.

**Conclusion:**

These are the first data available on the home range of domestic pigs kept under a free range system: the data show that pigs in these systems spend much of their time scavenging outside their homesteads, suggesting that these pigs may be exposed to infectious agents over a wide area. Control policies for diseases such as *Taenia solium*, Trypanosomiasis, Trichinellosis, Toxoplasmosis or African Swine Fever therefore require a community-wide focus and pig farmers require education on the inherent risks of keeping pigs under a free range system. The work presented here will enable future research to incorporate movement data into studies of disease transmission, for example for the understanding of transmission of African Swine Fever between individuals, or in relation to the life-cycle of parasites including *Taenia solium.*

## Background

Throughout the developing world the demand for meat products has been increasing by 4% per annum since the 1980s [1], and with continuing population growth this trend is unlikely to abate. The need for fast-maturing sources of animal protein, which require low cereal inputs places the non-ruminant animals in prime position for fulfilling this growing demand. To this end pig production is becoming increasingly popular, with pork and poultry contributing 76% of the increased meat consumption in the developing world between 1982–1998 [2].

**Figure 1:**
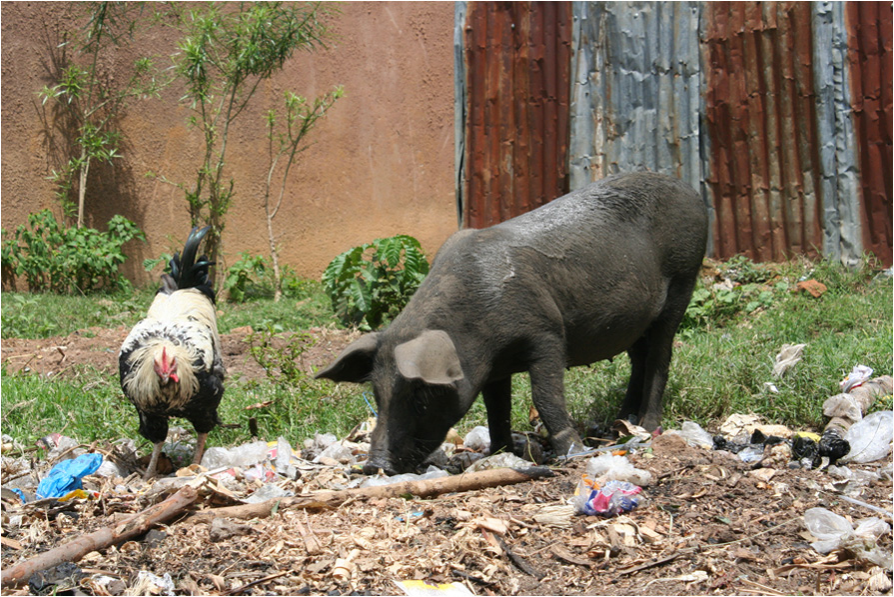
Free ranging pigs, near Busia, Western Kenya.

Pigs, *Sus scrofa*, have lower social prestige than cattle, but they are cheap to purchase and to raise and are therefore a popular option for resource-poor farmers, particularly women [3]. Taking advantage of the pig’s natural ability as a scavenger, many of these resource poor farmers opt for an extensive, low input form of production, whereby the pigs roam freely. These systems allow an animal to be kept without the need for expensive supplementary feedstuffs [4]. Pig production under these free range systems has been documented in many African countries, including: Kenya [5], Uganda [6], Tanzania [7], Cameroon [8] and Zambia [9]. Within our study area of western Kenya there is abundant evidence of this production system, as illustrated in Figure 1.

Pigs kept under all production systems can be the host of a variety of zoonotic and non-zoonotic pathogens, but allowing pigs to roam freely increases the disease transmission risk to the pig itself, to other wild and domestic animals, and to humans. Some diseases of particular relevance when considering free-roaming pigs are discussed below.

### Porcine cysticercosis

The zoonotic tapeworm, *Taenia solium,* is one of the leading causes of acquired epilepsy in the developing world [10]. The parasite has a two host life cycle, with humans as the definitive host, who become infected after consumption of viable cysticerci in under-cooked pork. The adult tapeworm inhabits the small intestine, causing an infection known as taeniasis, and gravid proglottids, containing thousands of infective eggs, detach from the adult worm and are excreted in faeces in an intermittent fashion [11]. Ingestion of these eggs, by either pigs or humans, results in the larval stage penetrating the intestinal wall and moving through the lymph and blood vessels to encyst in muscle, eyes or the central nervous system (CNS) as cysticerci [12].

As contact with infective human faecal material by pigs is a requisite for the successful propagation of the parasite lifecycle, it stands to reason that keeping pigs under a free-ranging system would increase the risk of the pigs acquiring this infection; this has been corroborated in several epidemiological studies [8,13-15].

### Trichinellosis

*Trichinella* spp. are tissue dwelling nematodes, which are transmitted to humans by the ingestion of undercooked meat containing infective larvae. The parasite has a wide range of mammalian hosts, but the majority of human infections are acquired through the consumption of pork, with European cases almost exclusively from outdoor or back-yard production systems [16]. Pigs acquire the infection through ingestion of infected wildlife carcasses, kitchen or slaughter waste. The ability of pigs to scavenge such material increases vastly when they are allowed to free range, heightening the relative risk of infection in comparison to confined pigs. The relative risk for *Trichinella* infection was estimated to increase by a factor of 25–100 times for free range pigs in comparison to pigs kept in indoor units [17].

### Toxoplasmosis

*Toxoplasma gondii* is a zoonotic protozoan parasite with a wide range of intermediate hosts, including pigs and humans, who acquire infection through the ingestion of infective oocysts excreted by cats, tachyzoites in raw milk, or encysted bradyzoites in infected meat [18]. The majority of human infections are thought to come from the ingestion of meat, in particular pork [19,20]. The risk of infection for a pig is again related to its ability to scavenge in areas contaminated with either cat faecal material containing oocysts, or carcasses containing infective bradyzoites; therefore, it is strongly associated with free-roaming behaviours.

Two studies from the Netherlands have found a significantly higher risk of seropositivity for toxoplasma antibodies in free range pigs than for those on an intensive pig unit [18,21]. Exposure to infective cat faeces or to infected carcasses in pigs raised outdoors are risks for disease transmission, which are likely to be exacerbated in the free range systems of the developing world.

### African swine fever (ASF)

ASF is a hemorrhagic virus of the Asfarviridae family, which has major epizootic potential [22]. This infection is characterised by high mortality in domestic swine. It is transmitted either by direct or in-direct contact between domestic pigs or wild suids with or without an arthropod vector and is maintained by three distinct cycles: 1) a sylvatic cycle between the Argasid tick and warthogs, and possibly bush pigs or giant forest hogs [23]; 2) a cycle between domestic pigs and the Argasid tick; and 3) a domestic pig cycle not requiring ticks [24]. There is also evidence that recently infected bushpigs and warthogs may be able to directly infect domestic pigs without need for the tick vector [23]. Wild boars have been implicated in virus transmission when they come into contact with infected free range domestic pigs, as was thought to be involved with the 2007 spread of ASF through Georgia [25]. Domestic pigs kept under free range systems are therefore at higher risk of contracting and transmitting ASF through contact with infected tick vectors or infected wild and domestic suids. Our study site in western Kenya has seen several ASF outbreaks over the last few years, most recently in 2011 [26].

### Trypanosomiasis

*Trypanosoma* spp*.,* transmitted by the tsetse fly (*Glossina spp.)*, cause a reduction in productivity in pigs and pose a high risk to human health, with *T. brucei gambiense and T. brucei rhodesiense* causing Human African Trypanosomiasis (HAT). The pig is a significant source of blood meals for the tsetse fly [27,28] and has been implicated in the epidemiology of both human and animal trypanosomiasis, with outdoor, free-roaming pigs being at particular risk of contact with tsetse flies. In particular, pigs have been identified as a significant reservoir of *T.b. rhodesiense* in our study site [29].

### Non-zoonotic helminths

Helminths, such as *Ascaris suum* and *Trichuris suis,* are responsible for substantial economic losses for pig producers throughout the world, through reduced weight gain, higher feed:gain ratio, condemnation of carcasses or organs and expenditure on prophylaxis or treatment [30]. *Ascaris suum* and *Trichuris suis* both require temperatures over 15°C for embryonation and larval development, and the prevalence of these parasites have been found to be higher in outdoor pig units than intensive, indoor units [31]. In a previous survey of free range pigs in the current study area pigs have been found to carry a substantial parasite burden, with an overall nematode prevalence of 84.2% and mean egg per gram (EPG) of 2,355 [32], which is likely leading to detrimental economic burden for their (often already poor) keepers.

To gain an understanding of the dynamics of disease within populations of free range pigs, the ecology of these animals must first be established. The behaviour of the domestic pig has been studied extensively within the context of intensive farming methods or through experiments to understand their social dynamics or learning ability [33,34]. Knowledge of domestic pig behaviour under free range conditions, specifically the size of the ‘home ranges’ and habitat preferences is, however, very limited with only one published paper from Mexico specifically looking to understand pig ecology under these systems [35] . The authors of this paper identified some interesting aspects of free-ranging pig behaviour, specifically in relation to coprophagia. What was lacking, however, was the quantification of ‘home range’ size and of habitat preferences of the pigs within this free range system, important elements of an understanding of the disease risks to which free range pigs are exposed.

The home range of an animal is “…that area traversed by the individual in its normal activities of food gathering, mating, and caring for young. Occasional sallies outside the area, perhaps exploratory in nature, should not be considered as in part of the home range” [36]. There are many different techniques available for determining the home range of animals and these have been extensively reviewed [37,38]. We utilise two such methods: minimum convex polygon (MCP) and local convex hull (LoCoH). The MCP is the simplest of the convex hull methods, which represents the smallest polygon with no inside angle greater than 180° that can be drawn to encompass all locations at which the animal was recorded. This is a simple measure to calculate and is used by the International Union for Conservation of Nature as the standard measure of a species home range [39]. The MCP method, however, is very sensitive to outlying points, which may reflect exploratory animal movement or measurement errors, providing an estimate of home range far beyond that utilised in the animal’s normal activities.

The k-1 nearest neighbours local convex hull technique was devised to improve on the MCP: it combines small MCPs which contain k-1 nearest neighbours, until all data points are included [40]. This technique has been shown to perform well to reduce type I (exclusion of utilised areas) and type II (inclusion of un-utilised areas) errors and is particularly useful in locations where geographical features provide hard boundaries to a home range. This method also allows the isopleths containing any percentiles of the data points to be identified, providing us with the ability to determine utilisation distributions for various percentiles of use, for example the 50% isopleth, which corresponds to the ‘core utilisation distribution’ and the 90% isopleth, corresponding to the true ‘home range’ [41].

There are several studies which investigate the home range of truly ‘wild living pigs’ [42], these being feral pigs of either domestic, European wild boar or hybrid origin. These studies have found a large variability in the home range (all based upon MCP determination) of these feral swine, from 0.52 km^2^[43] to 20.3 km^2^[44] for wild caught and released feral pigs. The large variability in roaming behaviours in these studies makes it difficult to extrapolate the findings outside of these particular study environments, potentially due to the impact of environmental features on the home range (e.g. proximity to human habitats, sharp ravines or cliff faces, forest cover, etc.). The environment that wild pig studies have encompassed are mainly forested or conservation areas, where the ability to move freely over large distances is greater and human interference is negligible. An extrapolation to the roaming behaviour of domestically bred and raised, albeit free-roaming, pigs would be highly inadvisable.

Here, we determine the geographical range of free-ranging domestic pigs in western Kenya, how far they travel during a day and night, and with which environmental features they spend time interacting.

## Methods

### Study area

**Figure 2:**
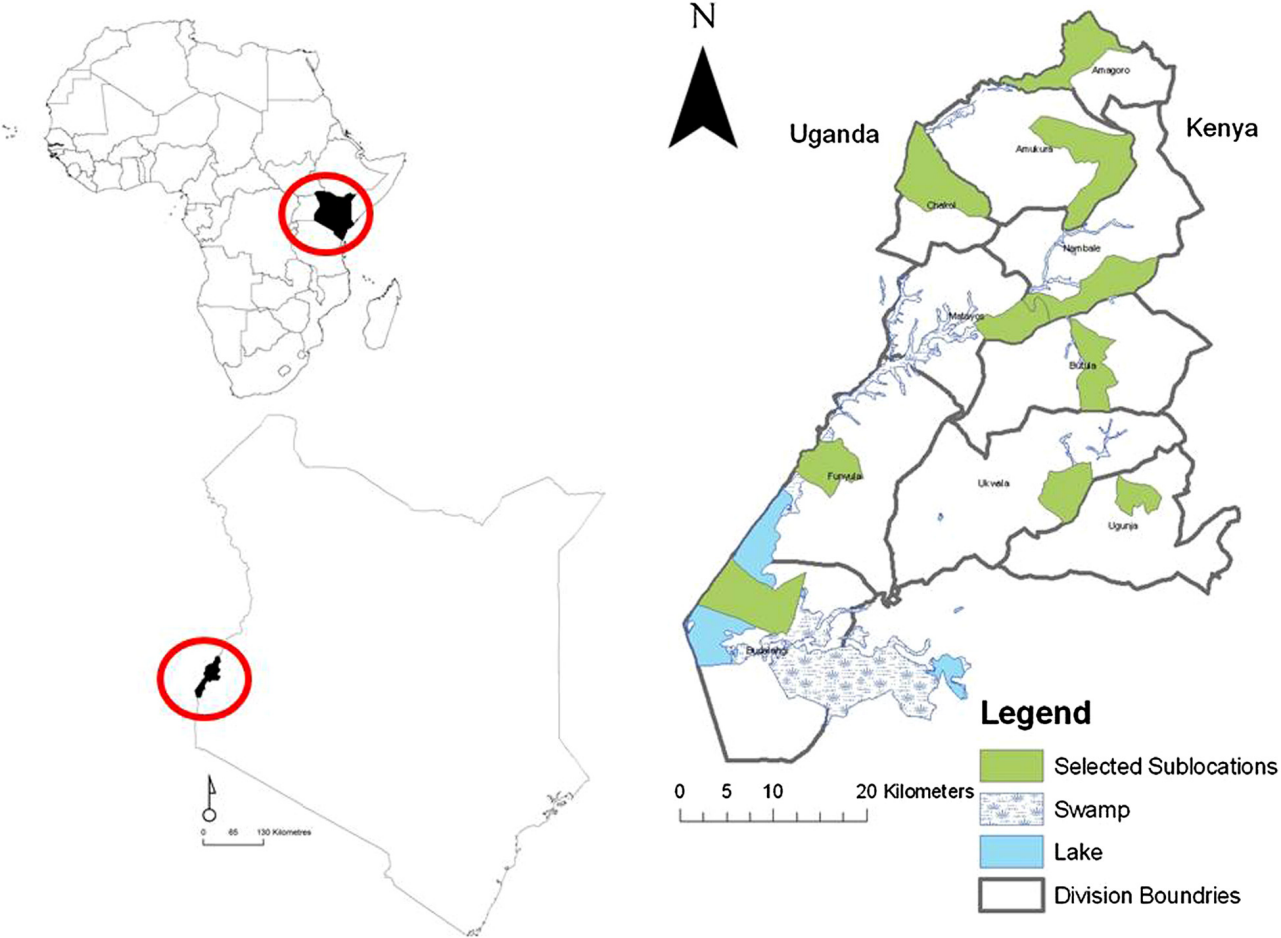
**Map of study area showing selected divisions: geographical data sourced from the ILRI GIS unit **[47]**with locator map showing location of study site in Kenya and of Kenya in Africa.**

The study area, shown in Figure 2, is representative of the Lake Victoria Crescent ecosystem. It falls within a 45 km radius of Busia town in western Kenya, bordered by Uganda to the west, Lake Victoria to the south, Mount Elgon to the north and Rift Valley Province to the east. The area is occupied predominately by members of the Luo, Luhya and Teso tribes. The area has bi-annual rains, occurring in March-May and August-October and supports a predominantly mixed crop-livestock production system with an average farm size of 0.5 ha [45]. Within this area, ten 3^rd^ level administrative units, called divisions, were selected based upon the popularity of pig production in these districts. Together these 10 divisions, Amagoro, Amukura, Budalangi, Butula, Chakol, Matayos, Funyula, Nambale Ujunga and Ukwala contain over 67% of the total pig population of the study area, which is estimated to be 66,307 by the district office of livestock and production: One sublocation (the smallest, 1^st^ level administrative unit) from each of these Divisions, was selected at random using the Hawths tools extension [46] for ArcMap 9.1 (ESRI, Redlands, USA). The ten selected study sub-locations,Bulemia, Anyiko, Asango, Sigalame, Nasewa, Bulwani, Malanga, Chakol, Amakuru and Kumuria can be seen in Figure 2.

### Animals

Between March 2011 and February 2012, one free range pig was randomly selected from each selected sublocation. The sample frame consisted of all pig keeping households within the sublocation, as provided by the relevant sublocation chief, a random number generator was used to pick the farmer from this list (farmers numbered first to last). On the selected farms pigs were excluded from the study if they were in the last trimester of pregnancy, were currently nursing piglets, were below 2 months of age or were due to be slaughtered in the next week (7 days from the day of selection). If more than one pig remained after exclusion they were allocated a number in age order and a random number generator was used to select the pig to be recruited, this was easy to achieve without any specific identifying procedure as the average pig herd size in the study site is only 2.6 (Unpublished Obs. EMF, LFD, EAC, WAdG). The study was explained to the farmer and their consent obtained before the animal was recruited into the study. The pigs were selected across the course of the year as only one GPS collar was available; the data were therefore obtained across different seasons.

### Data collection

A Garmin eTrex handheld GPS unit was used to obtain the coordinates of the homestead to which the pig belonged. The perimeter of the homestead, being that area utilised by the house for domestic activity (therefore excluding cropped fields), was tracked by walking along the boundary and if there was no discernible boundary the homestead members were asked for their best approximation of where their homestead perimeter lay. Features of the homestead (latrine, human dwelling, cooking point, rubbish disposal) were also mapped. A short questionnaire on pig husbandry was completed with the member of the homestead with the greatest involvement in the management of the pig.

**Figure 3:**
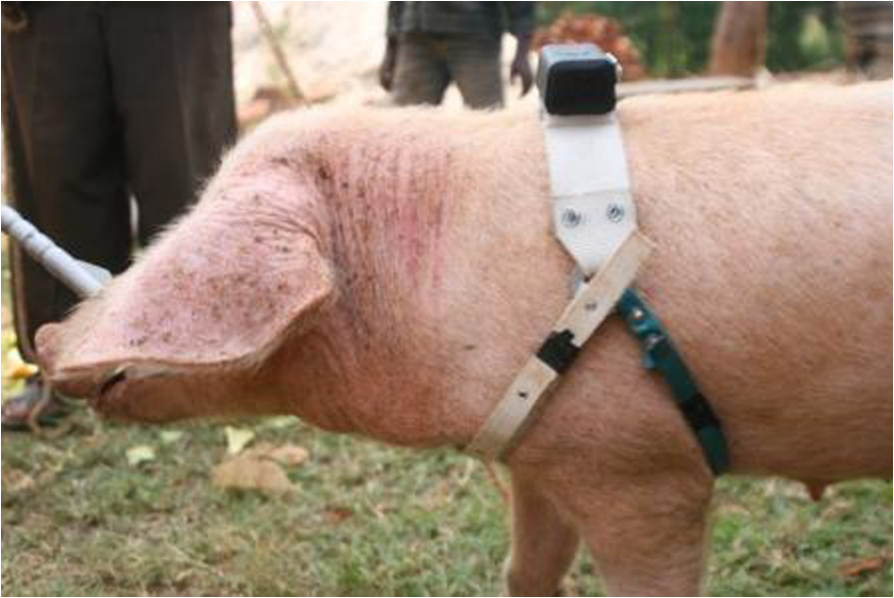
Pig restrained with a pig snare showing the correct fitting of the GPS unit.

The pig was restrained using a pig snare behind the upper canines and a lingual palpation to check for cysticercosis was performed [48]. Blood was collected from the external jugular or anterior vena cava into a 10 ml plain BD vacutainer® tube using an 18 gauge 1 ½ “ needle. A peripheral ear vein blood sample was collected using a blood lancet and micro-haematocrit tube and thick and thin blood smears were made immediately in the field. The pig was observed for the presence of ectoparasites and a note was made of the presence or absence of lice, mites or adult ticks, although ectoparasite species were not recorded. A faecal sample was taken from each pig and all biological samples were transported on ice to the Busia laboratory facility. A webbing collar fitted with a GPS unit and General Packet Radio Service (GPRS) data transmission system (Savannah Tracking Ltd, Nairobi, Kenya) was then fitted to the pig, as shown in Figure 3, and the pig released. The collar weighed ~350 g and operated using a 5400 mAmp/H rechargeable battery. Data were regularly uploaded to a server through the GPRS transmission system. The collar was set to record coordinates every 3 minutes for a one week (7 day) period from the day of recruitment.

Faecal samples were analysed for intestinal parasites using the McMasters [49] and Kato-Katz [50] methods. Thick and thin blood smears were stained with Giemsa and these smears were examined by microscopy for haemoparasites. Serum samples were analysed by HP10 Antigen ELISA [51] for the presence of viable *T. solium* infections.

### Analysis

Pig movement data from the GPS server were downloaded as a .csv file into Microsoft Excel and imported into ArcMap 9.1 and projected into UTM WGS 36 N. The LoCoh extension [52] for ArcGIS [40] was used to produce a utilization distribution of these data using the k-1 nearest neighbour local convex hull technique with 10 percentile isopleths. The value of K was determined by taking the square root of the number of GPS positions available as suggested by the software developers.

ArcMap 9.1 was then used to select the density isopleths representing both 50% (core utilisation distribution) and 90% (home range) of the points. A minimum convex polygon (MCP) was calculated using the Hawths Tools extension for ArcGIS. The Hawths Tools extension was then used to calculate the area of the layer files created from these selections, to create a track from the GPS movement data and to determine the length of that track.

Homestead points of interest and the perimeter boundary recorded using the handheld unit in the field were also imported into ArcMap 9.1. Individual points for each feature of a homestead and the perimeter boundary of each homestead (habitable area, as determined by the head of the household), were projected into UTM WGS 36 N and combined with the collar data to create informative data layers.

The area of the perimeter boundary polygon was calculated using Hawths Tools. The homestead features and the homestead itself were given a 5 m ‘buffer’ using Hawths Tools, 5 m being chosen to represent the accuracy of the GPS units used. All pig movement data points which fell within these buffer areas were selected and the time spent within the areas were calculated as a percentage of the total number of positions recorded for each pig.

All statistical analysis was performed using the ‘R’ language and environment for statistical computing [53]. The variables of interest were tested for violation of the assumption of normality using the Shapiro-Wilks test of normality, and due to the rejection of the null hypothesis (sample originating from a normally distributed population) for several of the variables it was decided to use non-parametric statistical methods, namely the Kruskal-Wallis rank sum test, Spearman’s Rank Correlation and the Wilcoxon signed rank test.

## Results

Ten pigs were selected and tracked during the time of this study, comprising 4 females, 2 male castrates and 4 male intact pigs with an average age of 6.7 months. All 10 pigs were kept under a free range system during the time of study. All pigs were fed supplementary food, being a combination of crop and household waste, with the household waste being fed uncooked to 8 of the 10 pigs.

No farmer reported any previous clinical episodes for any of the sampled pigs. Only 3 pigs had received any prophylactic treatments, which included Levamisole (1 pig), Deltamethrin (1 pig) and an unknown anthelminthic (1 pig). Reported anthelmintic treatment appeared to make no significant effect on the total nematode EPG (Kruskal-Wallis chi-squared = 2.7, p = 0.26). All pigs in this study were found to be infected by at least one parasite, with all pigs suffering from ectoparasites (adult ticks and lice in all cases) and 8 out of 10 also being infected with gastrointestinal parasites (*Strongyloides* spp, *Strongylus* spp., *Trichuris* spp., Coccidia and *Ascaris* spp. all being found). Three pigs were found to be infected with *Taenia solium* cysticercosis using the HP10 antigen ELISA [51,54] . A summary of the parasite burden for each pig is shown in Table 1. No haemoparasites were observed in any of the pigs.

**Table 1:** Summary of parasitic infections in study pigs

**Pig ID**	**Ectoparasite infection (lice and adult ticks in all)**	***Taenia Solium *Cysticercosis**	**Gastrointestinal parasites**	**EPG**	Total parasite spectrum*
**1**	**√**		Strongyles,	3,600	4
			Coccidia,	50	
			*Ascaris* spp.	13,900	
**2**	**√**		*Strongyloides* spp.	24	4
			Strongyles,	2,400	
			*Ascaris* spp.	700	
**3**	**√**	**√**			2
**4**	**√**	**√**	Strongyles	1,600	4
			*Ascaris* spp*.*	2,050	
**5**	**√**		*Strongyloides* spp.	50	*4*
			Strongyles,	48	
			*Ascaris* spp.	3,300	
**6**	**√**				1
**7**	**√**		Stongyles,	100	3
			Coccidia	250	
**8**	**√**		*Trichruis* spp.	100	3
			*Ascaris* spp.	5,650	
**9**	**√**		Strongyles	750	4
			*Trichuris* spp.	200	
			*Ascaris* spp.	400	
**10**	**√**	**√**	*Strongyloides* spp.,	750	*6*
			Strongyles,	350	
			Coccidia,	9,200	
			*Ascaris* spp	1,100	
**% pigs infected**	**100%**	**30%**	**80%**		

*Total parasite spectrum is defined as the total number of parasite species infesting each pig.

**Figure 4:**
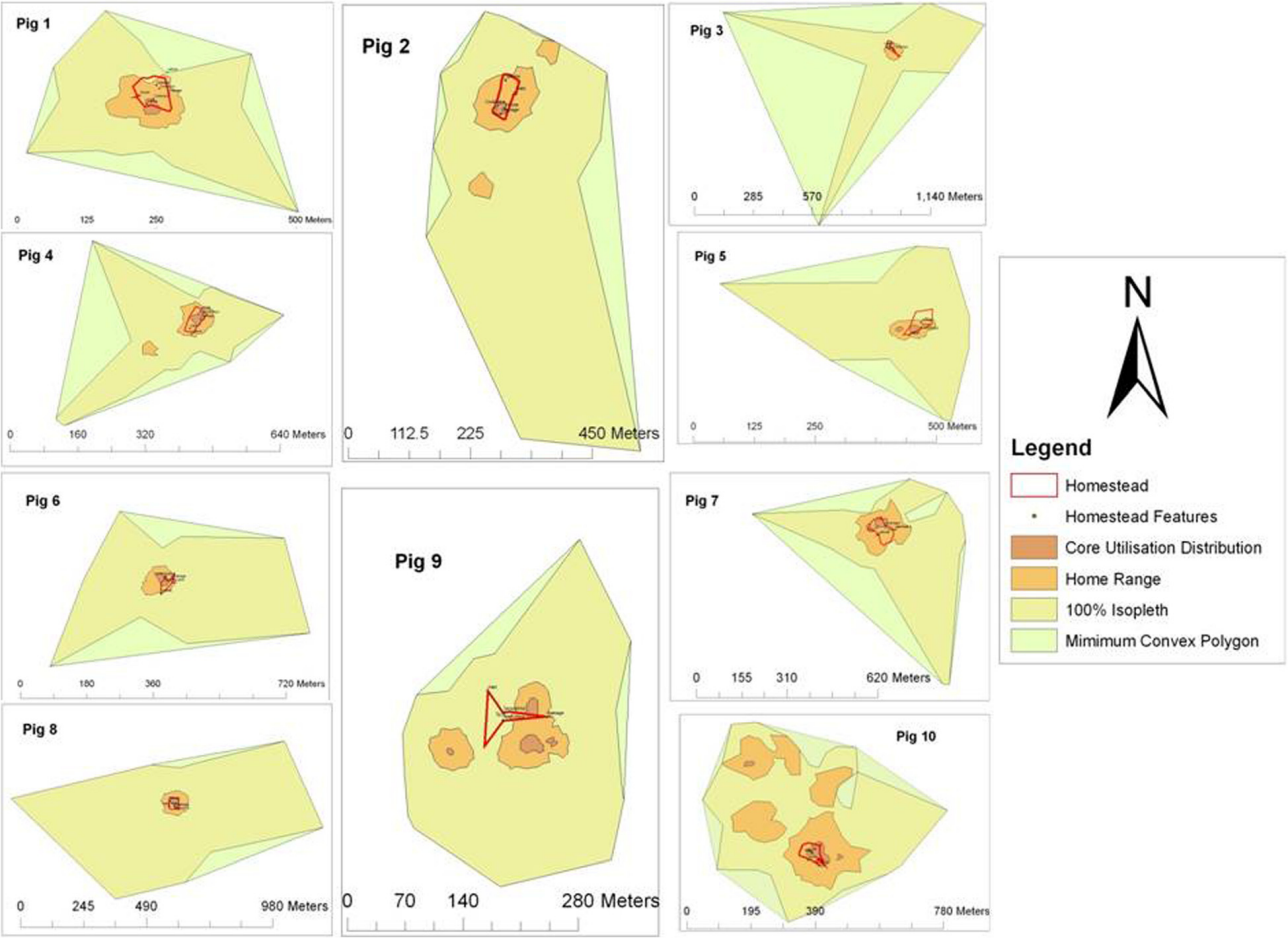
Illustration of movement parameters for each pig.

The minimum convex polygon, home range and core utilisation distribution were determined for each pig and are illustrated in Figure 4. The movement parameters calculated for each pig are also summarised in Table 2. The mean distance moved by a free range pig in our study site over a 12 hr period was 4,340 m, with pigs moving 4,169 m (range 1,401–6,383 m) during daylight hours and 4,511 m (range 1,293–7,809 m) at night, with no significant difference between these periods (Wilcoxon signed rank test w = 1 p = 1). The mean core utilisation distribution was found to be 947 m^2^ (range 133–3,353 m^2^) and the mean home range was found to be 15,085 m^2^ (range 2,937–74,887 m^2^).

**Table 2:** Pig movement data

**Pig ID**	**Ave. daily distance moved (m)**	**Ave. nightly distance moved (m)**	**Core utilisation distribution (m**^**2**^**)**	**Home range (m**^**2**^**)**	**MCP Area (m**^**2**^**)**	**Homestead area (m**^**2**^**)**	**% time spent within homestead perimeter**
1	1,401	1,293	612	9,315	108,617	224	54.1
2	3,707	4,067	409	12,685	346,585	2,143	70.7
3	3,824	3,479	424	5,380	709,809	1,048	61.6
4	3,463	3,387	133	5,805	123,189	1,707	51.1
5	2,992	2,815	410	2,937	101,650	1,666	65.7
6	4,557	4,812	701	4,993	197,420	775	34.7
7	5,933	7,809	1,582	19,554	267,869	4,328	61.7
8	6,383	6,927	967	7,540	429,339	1,646	66.7
9	4,608	4,293	873	7,749	81,218	802	8.55
10	4,825	6,219	3,353	74,887	289,990	2,834	53.8
Ave.	4,169	4,511	947	15,085	265,569	1,717	52.9

**Figure 5:**
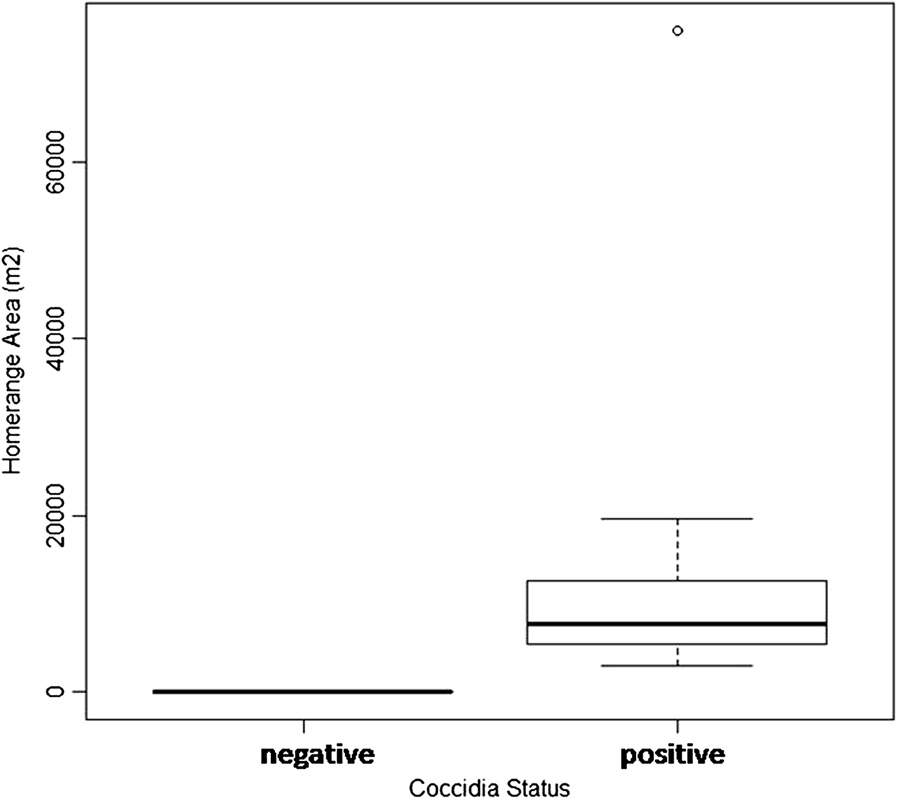
Boxplot of home range area and Coccidia EPG (rho = 0.75, p = 0.01).

In this small study, neither sex of pig or season were found to influence movement parameters as shown in Tables 3 &4 and no correlation was found between any movement parameter and the total parasite burden, calculated as sum of the eggs per gram (EPG) for all nematode species identified. No correlation was found either between movement parameters and the EPG of *Strongyloides* spp, Strongyles*, Trichuris* spp. and *Ascaris* spp, though a moderate correlation (Spearman’s rank correlation rho = 0.75, p = 0.01) was found between the home range area and the Coccidia EPG, though this was heavily influenced by an outlier as shown in Figure 5.

**Table 3:** Pig movement in dry and wet seasons

**Season**	**Daily distance moved (m)**	**Home range (m**^**2**^**)**	**Core Utilisation distribution (m**^**2**^**)**
‘Wet’ (pigs 3,4,5,6,7)	4,154	7,734	650
‘Dry’ (pigs 1,2,8,9,10 )	4,185	22,435	1,243
Kruskal-Wallis rank sum test	H = 0.2727 p = 0.6015	H = 3.153, p = 0.076	H = 0.8836 p = 0.347

**Table 4:** Pig movement according to sex

**Sex of Pig**	**Daily distance moved (m)**	**Home range (m**^**2**^**)**	**Core utilisation distribution (m**^**2**^**)**
Female (n = 4)	4,756	28,030	1,511
Male Castrate (n = 2)	2,613	7,348	518
Male Intact (n = 4)	4,362	12,015	596
Kruskal-Wallis rank sum test	H = 2.046, p = 0.36	H = 2.3727 p = 0.31	H = 1.146 p = 0.564

Pigs spent on average half (53%) of their time within the perimeter boundary of the households, or, otherwise stated, almost half their time outside the homestead. These homestead boundaries were often ill-defined, and all were porous. The time spent interacting within a 5 m radius of certain homestead features is shown in Table 5. The pigs in this study were shown to only spend on average 1.3% of their time interacting with the latrine area in their homestead of origin, 1.6% in the vicinity of the rubbish disposal area, 2.7% in the vicinity of the human dwellings and 4.3% in the vicinity of the cooking point: it is important to note that these interactions were only determined within the homestead of origin. Time spent interacting with homestead features was not found to influence parasite burden apart from in the case of *Ascaris* spp., where time spent interacting with latrines was found to be positively correlated with the EPG count (Spearman’s Rank Correlation rho = 0.81, p = 0.005).

**Table 5:** Interactions between pigs and homestead features

**Pig ID**	**Homestead area (m**^**2**^**)**	**% time spent within homestead perimeter**	**% time spend interacting with latrine**	**% time spend interacting with rubbish disposal**	**% time spend interacting with cooking point**	**% time spend interacting with human dwelling**
**1**	224	54.1	4.9**	0.2	2.5***	1.03
**2**	2143	70.7	0.1**	5.2	11.5*	6.8
**3**	1048	61.6	0.7**	Not observed	11.6**	5.9
**4**	1707	51.1	0.2**	4.1	0.9**	4.0
**5**	1666	65.7	0.8**	0.6	1.6*	2.0
**6**	775	34.7	0**	0.2	5.9*	2.8
**7**	4328	61.7	2.1**	0.1	0.1*	0
**8**	1646	66.7	3.7**	3.7	Not observed	3.4
**9**	802	8.55	0.03*	0.2	Not observed	0.1
**10**	2834	53.8	0.3*	0	0*	1
**Ave.**	1717	52.9	1.3%	1.6%	4.3%	2.7%

* Homestead feature fully enclosed.

** Homestead feature partially enclosed.

*** Homestead feature not enclosed.

## Discussion

This is the first study to have investigated the ecology of domestic pigs kept under a free range system, utilising GPS technology. We found that these pigs travel an average of 4,340 m in a 12 hr period and had a mean home range of 10,343 m^2^, within which the core utilisation distribution was found to be 964 m^2^. The lack of significant difference (p = 0.824) between day and night time movement indicates that the pigs are benefiting from a foraging strategy which involves both night and day scavenging. Nocturnal behaviour has been observed in wild pigs [55] who seem to be able to adjust their activity patterns based upon food availability [56].

Although this study was not designed to investigate population level influences on the movement parameters, it is interesting to note that no influence of season or sex of pig was found on any of the movement parameters. The pigs in this study were not influenced by management imposed restrictions on their movements during certain times of the year as selection criteria for the study animals was that they were kept on a free range basis. Another study in western Kenya that this team has conducted found only a 1.4% change in confinement in pigs between the wet and dry seasons (Unpublished Obs. LFT, EMF, EAC, WAdG).

No influence was found in this small study on parasite burden from movement parameters or interaction with homestead features apart from a positive correlation between *Ascaris* spp. EPG and the time spent interacting with latrines (Spearmans Rank Correlation rho = 0.81, p = 0.005) and a moderate positive correlation between Coccidia EPG and home range area (Spearmans rank correlation rho = 0.75, p = 0.01), the second of which appears to be highly influenced by one outlier. We could hypothesis that there may be a higher number of earthworms and dung beetles around a latrine area, which could be acting as paratenic hosts.

Despite the lack of association between the parameters measured and the health status of the pigs in this study, these findings do, however, have major implications for our understanding of pig husbandry and disease control within resource poor settings. For example, a domestic, free-ranging pig spends only ~50% of time within the homestead that owns it, indicating a high likelihood of exposure to environmental features, contaminants and pathogens outside the home area. Thus, when considering control policies for reducing infectious diseases in pigs, interventions targeting only pig owning households may be less effective than expected, and a community approach is clearly required.

Three out of the ten pigs recruited into this study were found to be positive for *T.solium* circulating antigen, which is a high prevalence compared to previous studies in the area which have found between 4% [32] and 10.5% [5]. However, a survey of 343 pigs at slaughter facilities in the study area immediately prior to the onset of the current study has found a prevalence of circulating antigen, using the same HP10 ELSIA of 55% (In prep. LFT, EMF, EAC, WAdG). This indicated that the area is, in fact, hyper-endemic for *T.solium* and we are therefore unsurprised that pigs selected on the basis of a known risk factor for cysticercosis infection were found to be infected.

In the case of *Taenia solium* cysticercosis, the porcine infection is acquired by the ingestion of infective eggs or proglottids in human faecal material that contaminates the pigs’ environment. Many studies have looked at the presence or absence of a latrine in a homestead as being a risk factor for cysticercosis infection in pigs; however, there has been no consensus between these studies. Some authors have found that the presence of a latrine is a risk factor for porcine cysticercosis [13,57] and others that latrines are protective [7,48,58]. In this study we found no association between the time spent interacting with a latrine on the homestead of origin and the *T.solium* status of the pig, which we believe suggests that the presence or absence of a latrine in an individual home is of less relevance to parasite transmission than overall provision of sanitation for the wider community in which the pig roams.

Although the observations made during this study suggest that pigs spend only a small amount of time interacting with the latrine area in their own homesteads (1.3%), we cannot discount the potential for pigs to come into contact with human faecal material elsewhere on the homestead or in neighbouring homesteads. We also note that any degree of access to human faecal material in or around a latrine, however short in time, is enough for transmission of the parasite to occur. Furthermore, 25% of homesteads in our study area do not have access to a latrine (In prep. EMF, LFD, EAC, WAdG), meaning that many people have no choice but to engage in open defecation, raising a very real possibility for pigs to contact human faecal material, and therefore potentially infective *T.solium* eggs. Finally, not all latrines are of the same quality, such that pigs may be able to access latrine buildings that are not properly enclosed: in this study area only 29% (in prep. LFT, EMF, EAC, WAdG) of latrine buildings were completely enclosed, and therefore not accessible to scavenging animals.

One method for the improvement of sanitation, which uses the whole community approach is the so called “community led total sanitation” [59]. This method attempts to trigger a community’s engagement with its own sanitation issues to reduce open defecation. Using this approach, communities take control of producing locally appropriate latrines and ensure that all community members use them. Such blanket coverage is likely to be far more effective than piecemeal individual adoption of latrines with respect to the exposure of free range pigs to faecal material.

Gastro-intestinal and ectoparasite infections are another important, production limiting issue for pig producers, as shown in Table 1. Heavy infestation with these parasites can lead to reduced weight gain in pigs [30], reducing the economic potential of these livestock. We found that only 2 of the 10 pigs recruited into this study were said to have had any anthelmintic in the 6 months prior to the study, and this was not found to have any influence on parasite load (in EPG for any nematode species). A lack of influence of levamisole treatment on EPG was also found in another study in western Kenya [32], suggesting either anthelmintic resistance, or incorrect usage of the drugs. Improved husbandry practises, including the use of effective anthelmintics at correct dosages, would enhance pig health and production in this study area. Importantly, we also find that the distances that free range pigs move on a daily basis (mean of 4.1 km during daylight and 4.5 km at night) are likely to entail high energy expenditure. Mature pigs 6–10 months old presenting at slaughter in this region have been found to have mean live weights at the abattoir of 30 kg, giving a dressed weight of only 22.5 kg and earning the farmer only 2,000–2,500 KES [60], equivalent to US$24–29 per animal. Encouraging the confinement of pigs is likely to improve feed conversion and weight gain, by both reducing un-necessary energy expenditure as well as limiting parasite burden through environmental exposure.

Confinement of pigs would also reduce the risk of contact with other domestic or wild pigs: pig to pig contact is a driver of African Swine Fever (ASF) virus transmission. ASF regularly causes outbreaks in this region, with two reported outbreaks at the end of 2010, both of which were reported as being resolved by early 2012 [26]. Confining pigs within correctly constructed pig stys would also reduce the chances of contact between pigs and tsetse flies [61] the vectors of *Trypanosoma spp*. Western Kenya is a trypanosomiasis endemic area and pigs are known to be important hosts and reservoirs [28,29].

Both Trichinellosis and Toxoplasmosis are very real threats to these free-ranging pigs, with access to kitchen waste, in particular meat products, being a risk factor for infection. Such swill is also implicated in ASF transmission. Pigs in this study were observed spending an average of 5.9% of their time in the vicinity of the cooking and waste disposal areas of their homestead of origin, illustrating the potential for ingestion of meat, which may contain infective tissue cysts of *Toxoplasma gondii* or *Trichinella spirallis*. Porcine toxoplasmosis can also be acquired through the ingestion of sporulated oocysts in cat faecal material: given that 49% of households in this region (unpublished obs.EMF, LFT, WAdG, EAC) report owning cats, combined with the scavenging behaviour of free range pigs, it is easy to infer from this the degree of contact with feline faecal material which takes place that may propagate this parasite.

While confinement would clearly be advantageous, there are practical and societal difficulties to overcome in encouraging the practice, not least because free range pig keeping is attractive to farmers due to the low input nature of the production system and the ease of implementation. Local extension services in areas where free ranging is practiced across East Africa should work to convince farmers that investing in improving pig production can reap important economic benefits in terms of weight at slaughter, as well as improve biosecurity and herd health on small-holdings.

## Conclusion

These data provide new insights into the behaviour of pigs kept under a free range system in a resource-poor setting. We believe that the data presented here can be used in conjunction with information on pig population densities to build contact network models and to better understand transmission of several pathogenic organisms. For example, understanding transmission of African swine fever between individual pigs or between domestic and wild pigs. The movement data can also be combined with information on ration formulation and daily weight gain to provide evidence-driven advice to farmers on how to change their animal husbandry practices to improve the profitability of pig production. The key messages are: 1) pigs kept under these systems spend almost half their time outside their homestead boundaries, such that the village environment beyond the farm matters just as much as the environment on the farm itself to pathogen transmission, and 2) free range domestic pigs expend tremendous energy foraging in the village environment, thus reducing their potential for weight gain and economic benefit to their owners.

## Abbreviations

ASF: African swine fever; ASFV: African swine fever virus; CNS: Central nervous system; EPG: Eggs per gram; GIS: Geographic information systems; GPS: Global positioning system; GPRS: General packet radio system; ILRI: International livestock research institute; LoCoH: Local convex hull; MCP: Minimum convex polygon.

## Competing interests

No author has any competing interest to declare.

## Authors’ contributions

LFT and EMF designed and implemented the study, and undertook data analysis. EMF obtained funding for the study. WAdG and EAC both assisted in the implementation of the study. All authors made contributions to conception, design, and revision of the manuscript. All authors read and approved the final manuscript.
